# Is Penicillin Plus Gentamicin Synergistic Against Sessile Group B Streptococcal Isolates? An *in Vivo* Study With an Experimental Model of Foreign-Body Infection

**DOI:** 10.3389/fmicb.2018.00919

**Published:** 2018-05-15

**Authors:** Corinne Ruppen, Thomas Mercier, Denis Grandgirard, Stephen L. Leib, Cristina El Haj, Oscar Murillo, Laurent Decosterd, Parham Sendi

**Affiliations:** ^1^Institute for Infectious Diseases, Bern University Hospital, University of Bern, Bern, Switzerland; ^2^Graduate School for Cellular and Biomedical Sciences, University of Bern, Bern, Switzerland; ^3^Service and Laboratory of Clinical Pharmacology, Department of Laboratories, Lausanne University Hospital, Lausanne, Switzerland; ^4^Laboratory of Experimental Infection, Infectious Diseases Service, Bellvitge Biomedical Research Institute–Hospital Universitari Bellvitge, Barcelona, Spain

**Keywords:** *Streptococcus agalactiae*, biofilms, penicillins, gentamicin, synergism, foreign bodies

## Abstract

The rate of invasive group B *Streptococcus* (GBS) infections is steadily increasing, particularly in older persons and in adults with diabetes and other comorbidities. This population includes persons with a foreign body (e.g., who have undergone arthroplasty). In a rat tissue cage model, we evaluated the efficacy of adjunctive gentamicin (GEN) administered systemically (5 mg/kg body weight) every 24 h, or locally (12.5 mg/L tissue cage concentration) every 24 or 72 h, in combination with penicillin (PEN) administered systemically (250,000 IU/kg body weight three times per day). The efficacy was evaluated on two different sessile forms of GBS: transition (i.e., in between planktonic and biofilm) and biofilm. After 3 days of treatment, the mean bacterial load reduction of transition-form GBS was greater in all PEN–GEN combination groups than in the PEN monotherapy group (*P* ≤ 0.03). The 6-day regimen decreased the bacterial load significantly in comparison to the 3-day regimen, irrespective of growth form and adjunctive GEN (*P* < 0.01). After 6 days of treatment, the mean reduction in transition-form GBS was greater with PEN plus GEN administered locally every 24 h than with PEN monotherapy (*P* = 0.03). These results were not confirmed with biofilm GBS. The difference in mean bacterial load reduction between all PEN–GEN and PEN monotherapy groups was <100 CFU/mL. Hence, synergy criteria were not fulfilled. Adjunctive systemic GEN consists of potential side effects and showed poor efficacy in this study. Combining systemic PEN and local GEN has a potential application in the treatment of streptococcal implant-associated infections.

## Introduction

The rate of invasive group B *Streptococcus* (GBS, *Streptococcus agalactiae*) disease in non-pregnant adults has substantially increased in the past decade (Skoff et al., 2009; Ballard et al., 2016). In particular, older persons and adults with diabetes and other substantial comorbidities are at risk (Sendi et al., 2008; Skoff et al., 2009). For GBS periprosthetic joint infection (PJI) and infective endocarditis, a combination therapy of penicillin G (PEN) plus gentamicin (GEN) for the first 2 weeks followed by PEN monotherapy is recommended (Zimmerli et al., 2004; Westling et al., 2007). The rationale for this combination therapy is a postulated synergistic effect (Schauf et al., 1976; Deveikis et al., 1977; Cooper et al., 1979; Baker et al., 1981). In contrast to these study findings, the results of our recent experiments did not confirm a synergistic effect with planktonic GBS (Ruppen et al., 2016b, 2017a). In view of the potential nephrotoxicity of aminoglycosides and the increasing elderly population at risk for invasive GBS disease, the decision to administer or withhold aminoglycosides is of clinical relevance. Given the fact that in PJI, sessile forms of bacteria are involved, we evaluated whether this combination therapy is active against biofilm GBS *in vitro* (Ruppen et al., 2016a). Although a synergistic effect was seen in two of four tested GBS isolates, the results indicated that the required GEN concentration cannot be achieved in an extravascular compartment with systemic administration (Ruppen et al., 2016a). The translation of these results into *in vivo* conditions requires caution. The effect of concentration fluctuation is not reflected *in vitro*, since fixed antibiotic concentrations are used. In addition, in infections affecting tissues or biological fluids, the pH can be acidic, and this milieu increases the minimum inhibitory concentrations (MICs) of aminoglycosides for Gram-positive cocci (Baudoux et al., 2007). Therefore, the objective for the present *in vivo* study was to test the synergistic effect of GEN – administered either systemically or locally – as an adjunct to systemic PEN treatment in an experimental model of GBS foreign-body infection. In consideration that elderly people are at risk for both invasive GBS and adverse events from antimicrobial treatment, we focused on aged animals in the model.

## Materials and Methods

### Ethics Statement

The Animal Care and Experimentation Committee of the Canton of Bern, Switzerland, approved this study (License and Reference No. BE 80/16). According to the Swiss national guidelines for the performance of animal experiments, a recovery phase must follow an intervention phase (e.g., day or light cycle for 12 h). During the recovery phase, neither diagnostic nor therapeutic interventions are allowed.

### GBS Strain

The isolate was obtained from a patient with PJI (BE07-1b) and characterized previously (serotype Ib, multilocus sequence type 8, PEN MIC 0.032 mg/L, GEN MIC 24 mg/L) (Ruppen et al., 2016a,b, 2017a).

### Animals

Female retired breeder Wistar Han rats purchased from Charles River Laboratories (Sulzfeld, Germany) were used in all experiments. Median weights of animals are indicated in the corresponding sections below. The rats were housed in groups of four that had previous surgical procedures and singly after the operation to prevent implant removal due to social behavior. They were housed in individually ventilated cages with unlimited access to food and water. Room temperature was maintained (22 ± 2°C) with a 12-h light/dark cycle.

### Tissue Cage Infection Model

Tissue cages were implanted in animals, as reported previously (Zimmerli et al., 1982; Murillo et al., 2006). In brief, two teflon tissue cages with 150 spaced perforations of 1-mm diameter (Mecanizados del Besos, Badalona, Spain) were subcutaneously (s.c.) implanted. Each tissue cage contained two polymethylmethacrylate coverslips (Mecanizados del Besos, Badalona, Spain) (see Supplementary Figure S1). Eighteen days after implantation of tissue cages (i.e., recovery time after surgery), tissue cage fluid (TCF) was examined for sterility (see Supplementary Figure S2). Upon documentation of no bacterial growth in TCF, pharmacokinetic (PK), and GBS infection studies were started.

### PK Studies

The median weights of animals included in the PK studies were 370 g (number of animals: 21 and weight range: 275–548 g). Antibiotic concentrations were measured in animals without introducing an infection. At each measured time point, three samples from three different animals were obtained. The time interval in obtaining two consecutive TCF samples was never <6 h in any animals (to minimize their stress). The PK study included three treatment regimens:

(i) PEN 150 mg/kg (250,000 IU/kg) body weight (BW) given three times systemically (i.e., s.c.) with a 4-h interval between each administration (number of animals: 9). The dose was chosen on the basis of a previous PEN PK study in rats (Gavalda et al., 1997). The administration was restricted to three doses because of the 12-h-cycle intervention restriction (see section “Ethics Statement”).
(ii) GEN 5 mg/kg BW s.c. as a single dose (number of animals: 9).
(iii) GEN locally, final tissue cage concentration 12.5 mg/L (tissue cage volume 2.5 mL, injection volume 0.2 mL with GEN 156 mg/L; number of animals: 3).

The rationale for selecting these PEN and GEN doses was based on reported levels of corresponding antibiotics found in human serum (Kim and Bayer, 1985; Kim, 1987; Gavalda et al., 1997) and our previous *in vitro* study with biofilm GBS (Ruppen et al., 2016a). In animals treated with regimens (i) and (ii), TCF samples were obtained at the following time points: 0.5, 1, 4, 6, 8, 12, and 24 h. After 24 h, animals were euthanized and a serum sample obtained. In animals treated with local GEN (group iii), TCF and serum samples were obtained at the following time points: 24, 48, and 72 h. Thereafter, animals were euthanized (see Supplementary Figure S2).

### GBS Infection Study

The study plan included 32 animals (median weight: 359.5 g and range: 268–552 g). Thirty-one rats (one loss after anesthesia) were infected with an inoculum of 2–5 × 10^4^ CFU/mL in 0.1 ml injected into the tissue cage. Four days later, TCF was extracted to quantify the bacterial load in the tissue cage (i.e., pretreatment colony count) (**Figure 2**). Thereafter, antimicrobial treatment was started. The systemic antimicrobial treatment duration was either 3 or 6 days (see Supplementary Figure S2). The compound in the negative control group consisted of 0.9% sodium chloride (Sintetica, Mendrisio, Switzerland). Because acidic pH can increase MICs of aminoglycosides for Gram-positive cocci (Baudoux et al., 2007), the pH in TCF was measured at days 1, 3, and 6 with indicator stripes (Macherey-Nagel, Düren, Germany).

### Antimicrobial Agents and Serum Concentration Measurements

PEN (benzylpenicillin-sodium, Grünenthal Pharma AG, Mitlödi, Switzerland) and GEN (Hexal AG, Holzkirchen, Germany) were supplied from the clinical pharmacy of the University Hospital (Bern, Switzerland). Prior to administration, antibiotic concentrations in original vials were diluted until they reached the targeted value and were then measured, as described previously (Ruppen et al., 2016b). Antibiotic concentrations of PEN and GEN in serum and TCF samples obtained from animals were measured via high-performance liquid chromatography-mass spectrometry(Ruppen et al., 2016b).

### Systemic Treatment and Combination Thereof

For comparative analyses, the efficacy of PEN monotherapy was evaluated with that of PEN–GEN combination therapy. PEN monotherapy consisted of PEN G 150 mg/kg BW s.c. as three doses daily (every 4 h followed by an antibiotic-free interval of 12 h). The administration was restricted to three doses per day because of the 12-h-cycle intervention restriction (see section “Ethics Statement”). The combination therapy consisted of PEN as described for monotherapy plus GEN 5 mg/kg BW s.c. as one dose per day (see Supplementary Figure S2).

### Combination of Systemic PEN Plus Local GEN Treatment

These combinations consisted of PEN, as described earlier, plus GEN 12.5 mg/L injected into the tissue cage as one dose either every 72 h or every 24 h. The former regimen was evaluated after 3 and 6 days, the latter only after 6 days (see Supplementary Figure S2).

### Read-Out and Synergy Definition

The efficacy of the treatment regimen on sessile bacteria in transition form was evaluated in samples obtained from TCF after 3 and 6 days of treatment. Samples were plated on Columbia sheep blood agar and incubated at 37°C in 5% CO_2_ for 24 h for colony counting. The efficacy of the treatment regimen on sessile bacteria in biofilm was evaluated in samples obtained from coverslips after 3 and 6 days of treatment. Animals were euthanized and coverslips removed from tissue cages and subjected to trypsin and sonication, as described previously (Murillo et al., 2006; Ruppen et al., 2016a). Dislodged bacteria were then plated on Columbia sheep blood agar and incubated at 37°C in 5% CO_2_ for 24 h for colony counting.

The synergy definition was modified according to the experimental setting and defined as a ≥100-fold (≥2 logs) increase in killing after 3 and 6 days of treatment (as measured by colony counts [CFU/mL]) with the PEN plus GEN combination therapy in comparison with the PEN monotherapy.

Graphical representation and statistical analyses were performed with GraphPad Prism 7.01 (GraphPad Software, Inc., San Diego, CA, United States). A *P-*value of < 0.05 calculated by the Mann–Whitney test was considered significant.

The efficacy of antibiotics was evaluated on two bacterial growth forms. The first form was obtained from TCF and these bacteria were regarded as GBS in a “transition form” from a planktonic to a biofilm state and vice versa (see Supplementary Figure S1). The second form was obtained from sonicated fluid after coverslips were sonicated and treated with trypsin. These bacteria were regarded as “biofilm bacteria” (see Supplementary Figure S1).

## Results

### PK Studies

**FIGURE 1:**
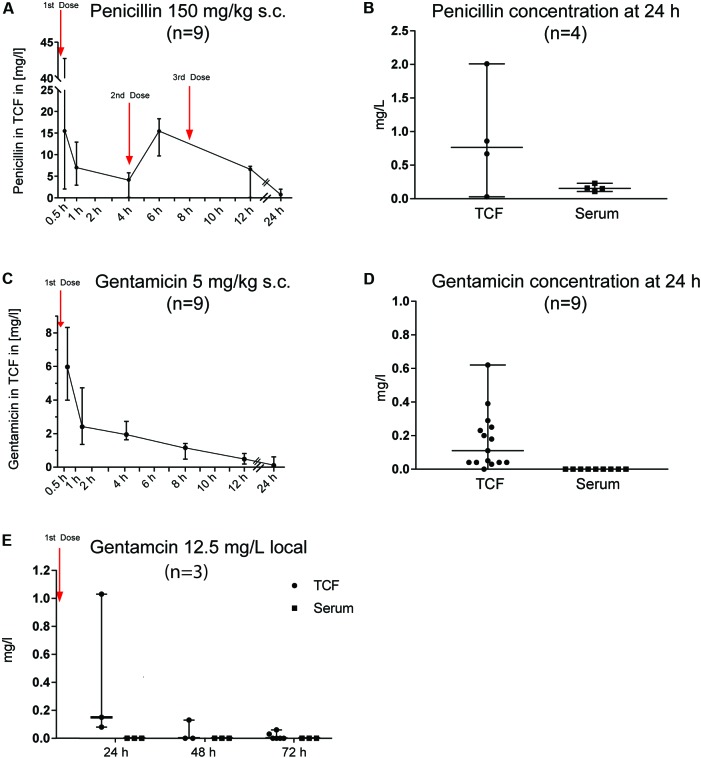
Pharmacokinetics of penicillin G (PEN) and gentamicin (GEN) in tissue cage fluid (TCF) and serum. The numbers in brackets reflect the total number of animals used for the PK study. At each measured time point, three samples from three different animals were obtained. The time interval in obtaining two consecutive TCF samples was never <6 h in any animals (to minimize their stress). Results are presented as median ± range. **(A)** PEN 150 mg/kg body weight (BW) subcutaneously (s.c.) given three times (every 4 h). **(B)** PEN 150 mg/kg BW s.c. given three times (every 4 h) and measured in TCF and serum after 24 h. **(C)** GEN 5 mg/kg BW s.c. given once daily. **(D)** GEN 5 mg/kg BW s.c. given once daily and measured in TCF and serum after 24 h. **(E)** GEN 12.5 mg/L applied locally once and the concentration measured in TCF and serum after 24, 48, and 72 h.

The systematically administered antimicrobials rapidly penetrated into the tissue cage. Thirty minutes after administration, the median PEN and GEN concentrations were 15.49 and 5.97 mg/L (range: 2.04–42.74 and 3.99–8.33 mg/L), respectively. The PKs of PEN and GEN concentrations in the TCF are illustrated in **Figures 1A,C**. Because of obtaining repetitive TCF samples (**Figure 1A**), measurement of penicillin in TCF at the 24-h time point was possible in only four animals; serum concentrations corresponding to the TCF sample are displayed in **Figure 1B**. Twelve hours after the last dose (i.e., 24 h after the first dose), the median PEN concentration in the TCF dropped to 0.765 mg/L (range: 0.03–2.01), but was still above the PEN MIC of the GBS isolate (0.032 mg/L). At 24 h, the corresponding median PEN concentration in serum was 0.155 mg/L (range: 0.11–0.23) (**Figure 1B**). Twenty-four hours after a single dose of systemically administered GEN (5 mg/kg BW), the median concentration in the TCF was 0.11 mg/L (range: 0–0.62), while it was undetectable in serum (**Figure 1D**). At 24, 48, and 72 h after a single dose of locally administered GEN (12.5 mg/L), the TCF concentration dropped to ≤1 mg/L and was not measurable in the serum at any corresponding time points (**Figure 1E**).

### GBS Infection Study

Four of 31 animals were excluded from the results because of contamination of the tissue cage with additional microorganisms. Hence, the results for 27 animals were available for the read-out. None of the animals developed sepsis or died from infection.

#### pH in the TCF During the Infection Study

At days 1, 3, and 6, the pH in the TCF remained stable at >6 and <7.5, indicating that the pH of the milieu had no considerable effect on the GEN MIC of GBS.

#### Effect of Antimicrobial Treatment on Sessile Bacteria in Transition Form

##### Systemic therapy, 3 days of treatment (**Figure 2A**)

The mean bacterial load was significantly lower in the PEN–GEN group (4.9 [SD ± 5.7] × 10^5^ CFU/mL) than it was in the corresponding PEN monotherapy group (2.3 [± 3.5] × 10^6^ CFU/mL) (*P* = 0.031). Because the difference was <100 CFU/mL, criteria for synergism were not fulfilled.

##### Systemic therapy, 6 days of treatment (**Figure 2B**)

The bacterial load was significantly lower after 6 days of PEN monotherapy (2.2 [± 4.4] × 10^5^ CFU/mL) than it was after all 3-day treatment regimens (*P* = 0.0007). Within the groups with 6 days of treatment, the difference in the mean bacterial load with PEN monotherapy was not significant in comparison to that of PEN–GEN combination therapy (0.9 [± 1.2] × 10^4^ CFU/mL).

##### Systemic PEN plus local GEN therapy, 3 days of treatment (**Figure 2C**)

The combination of PEN plus local GEN treatment showed a significant reduction in the mean bacterial load to 4.9 [± 7.1] × 10^5^ CFU/mL (one GEN dose every 72 h) and 1.4 [± 1.0] × 10^5^ CFU/mL (one GEN dose every 24 h), respectively, in comparison to that of systemic PEN monotherapy (2.3 [± 3.5] × 10^6^ CFU/mL) (*P* = 0.03 and *P* < 0.01, respectively).

##### Systemic PEN plus local GEN therapy, 6 days of treatment (**Figure 2D**)

When local GEN was administered every 24 h, the mean bacterial load reduction was significant in the combination group in comparison to that in the PEN monotherapy group (5.8 [± 2.9] × 10^3^ CFU/mL versus 2.2 [± 4.4] × 10^5^ CFU/mL) (*P* = 0.03). The difference was not significant if GEN was administered every 72 h.

#### Effect of Antimicrobial Treatment on Sessile Bacteria in Biofilm

##### Systemic therapy, 3 days of treatment (**Figure 3A**)

There was no considerable difference in bacterial load when PEN monotherapy was compared with systemic PEN–GEN combination therapy.

##### Systemic therapy, 6 days of treatment (**Figure 3B**)

The mean bacterial load was significantly reduced after 6 days of PEN monotherapy (0.8 [± 1.4] × 10^4^ CFU/mL) in comparison with that after 3-day treatment with monotherapy (2.6 [± 4.1] × 10^5^ CFU/mL) (*P* < 0.01). However, there was no significant difference in bacterial load when PEN monotherapy was compared with systemic PEN–GEN combination therapy after 6 days.

##### Systemic PEN plus local GEN therapy, 3 days of treatment (**Figure 3C**)

No significant difference in bacterial load was seen when PEN monotherapy was compared with PEN plus single-dose local GEN (i.e., one dose for 72 h) combination therapy.

##### Systemic PEN plus local GEN therapy, 6 days of treatment (**Figure 3D**)

The most pronounced reduction in the mean bacterial load to 7.4 [± 8.6] × 10^2^ CFU/mL (i.e., 1.6 [± 0.9] × 10^6^ CFU/mL reduction in comparison to that in the control group) was observed with PEN plus one dose of local GEN given every day. Interestingly, the mean bacterial load with PEN monotherapy was significantly lower than that with PEN plus local GEN combination therapy (one dose every 72 h). There was a considerable range in sample results (1 × 10^2^–5 × 10^4^ CFU/mL PEN monotherapy versus 5 × 10^3^–1 × 10^6^ CFU/mL PEN plus local GEN every 72 h).

## Discussion

In GBS foreign-body infections in humans, PEN is commonly administered intravenously every 4 to 6 h (e.g., 18–24 million U/day) (Zimmerli et al., 2004). There are, however, few PK studies on PEN concentration in extravascular compartments (e.g., synovial fluid or bone). The mean PEN serum concentration is approximately 80 mg/L 30 min after intramuscular administration and 3 mg/L 4 h after completion of intravenous administration of 5 million IU PEN (Plaut et al., 1969; Geddes and Gould, 2010; Grünenthal Pharma AG, 2012). The elimination half-time of PEN is 30 min –in both humans and rats (Gavalda et al., 1997) – and the penetration in synovial fluid is reported to be ≥50% of serum levels (Parker and Schmid, 1971; Grünenthal Pharma AG, 2012). Hence, from mathematical extrapolation, the expected PEN concentration in synovial fluid is approximately 1.5 mg/L 4 h after completion of intravenous administration of 5 million IU PEN. The recommended dosage for adjunctive GEN treatment in humans is 3 mg/kg BW given intravenously, leading to a serum peak concentration of 12 to 14 mg/L. GEN penetrates well to synovial fluid (i.e., >50% of serum levels) (Dee and Kozin, 1977). It has an average serum elimination half-time of 2.5 h in humans and <1 h in rats (Tran Ba Huy et al., 1986). In analogy to the above-mentioned extrapolation, approximately 6 to 7 mg/L peak concentration and <1 mg/L after 24 h is estimated in synovial fluid. Similar PEN and GEN values were targeted in our *in vivo* model, though it is unknown whether the antibiotic concentration in synovial fluid corresponds to that in TCF. In addition, the mathematical extrapolation may be imprecise given the many confounders in a biological system and the different routes of administration. However, the PK results in the TCF reflect those in an extravascular compartment, and the drug levels after 24 h in both serum and TCF were within the expected range (**Figures 1A–E**).

**FIGURE 2:**
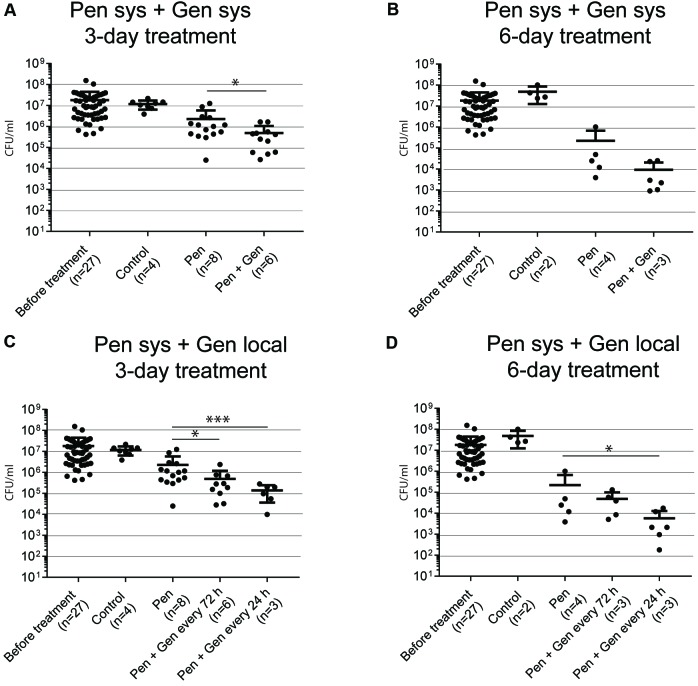
Bacteria in transition form measured in TCF after 3 and 6 days of treatment. The numbers in brackets below the *x*-axis reflect the number of animals (each animal with two tissue cages). Each dot reflects one sample. **(A)** GBS CFU/mL after 3 days of systemic (sys) penicillin (PEN) and penicillin plus gentamicin (GEN) treatment. **(B)** GBS CFU/mL after 6 days of systemic PEN and PEN plus GEN treatment. **(C)** GBS CFU/mL after 3 days of systemic PEN and local GEN treatment. **(D)** GBS CFU/mL after 3 days of systemic PEN and local GEN treatment. Data represent mean ± standard deviation (SD). Statistical analysis was calculated with the Mann–Whitney test, representing a value of ^∗^*P* < 0.05 and *P* < 0.001.

**FIGURE 3:**
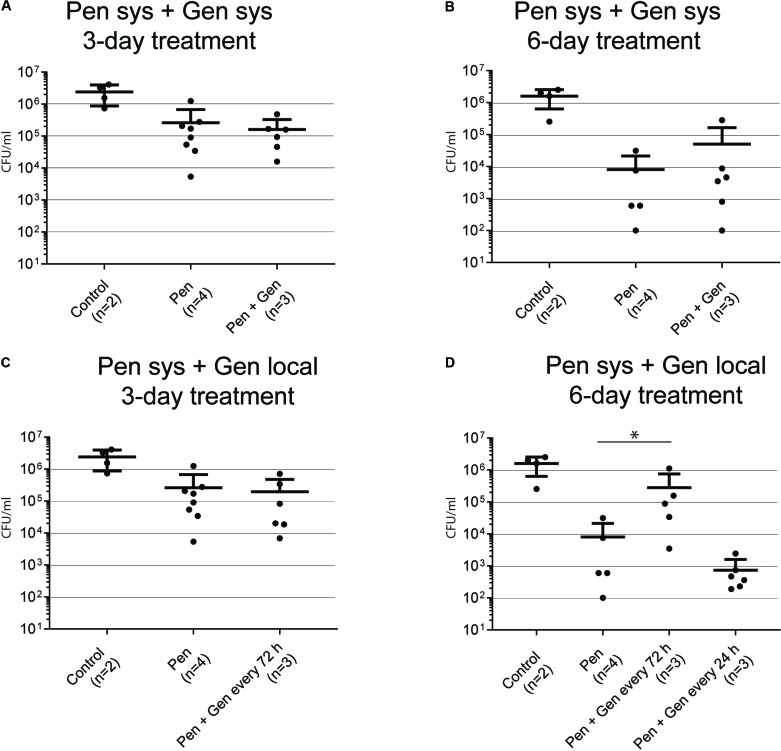
Bacteria in biofilm recovered from coverslips after 3 and 6 days of treatment. The numbers in brackets below the *x*-axis reflect the number of animals (each animal with two tissue cages). Each dot reflects one sample. **(A)** GBS CFU/mL after 3 days of systemic (sys) penicillin (PEN) and penicillin plus gentamicin (GEN) treatment. **(B)** GBS CFU/mL after 6 days of systemic PEN and PEN plus GEN treatment. **(C)** GBS CFU/mL after 3 days of systemic PEN and local GEN treatment. **(D)** GBS CFU/mL after 6 days of systemic PEN and local GEN treatment. Data represent mean ± SD. Statistical analysis was calculated with the Mann–Whitney test, representing a value of ^∗^*P* < 0.05.

Significant bacterial growth was detected in samples cultured from TCF and coverslips despite PEN concentrations being above the GBS MIC during the entire treatment period. This points toward the foreign-body infection treatment concept of considering the minimal biofilm eradication concentration (MBEC), which is substantially higher than the PEN MIC (Ruppen et al., 2016a). We analyzed two forms of sessile bacteria because biofilm biology is regarded as a continuum of various growth phases (i.e., from planktonic to stationary and vice versa) (see Supplementary Figure S1). The reduction in bacterial load was minor after 3 days of treatment, irrespective of growth form and treatment regimen. At this measured time point, we observed – in line with our previous *in vitro* results (Ruppen et al., 2016b) – a significant difference in killing of transition-form GBS between PEN monotherapy and systemic PEN–GEN combination therapy, without fulfilling the criteria for synergism (**Figure 2A**). The difference between PEN monotherapy and systemic PEN–GEN combination therapy was not observed for assays with biofilm bacteria and not after 6 days of treatment. Six days of PEN treatment was significantly more efficient than 3 days of PEN treatment in reducing the bacterial load, but it did not cure the infection, irrespective of adjunctive GEN treatment. This confirms that short treatment duration is insufficient for foreign-body infections. It also indicates that prolonged PEN treatment may have an effect on biofilm bacteria (**Figure 3D**), even when MBECs are higher than MICs by a considerable magnitude (Ruppen et al., 2016b). Drug accumulation in the tissue cage may have contributed to the bacterial killing. However, we did not assess PEN concentration accumulation in TCF after 6 days. Synergism was not observed in any of the assays in which PEN monotherapy was compared with systemic PEN–GEN combination therapy. We have previously outlined that, with systemic administration, the required GEN concentration in the extravascular compartment may not be reached (Ruppen et al., 2016a). Indeed, 30 min after systemic administration, GEN concentrations in TCF ranged from 4 to 8 mg/L and dropped rapidly thereafter. This PK pattern was also seen when the same formulation with a concentration of 12.5 mg/L was administered locally every 72 h. A slow-release drug formulation with high GEN concentration for local treatment may be a better treatment option (Humphrey et al., 1998). In line with this reasoning and the results found in our *in vitro* studies with biofilm GBS (Ruppen et al., 2016a), the most pronounced killing for biofilm bacteria was observed when systemic PEN and local high-dose GEN was used every 24 h for a prolonged treatment period (**Figure 3D**).

The mechanism of action of adjunctive GEN against non-replicating bacteria is poorly understood. It is possible that – at least in part – the GEN MIC, and hence the GEN MBEC, play a more important role than they do in planktonic bacteria (Ruppen et al., 2017b). In four biofilm GBS with GEN MICs ranging from 8 to 32 mg/L and MBECs from 8 to 64 mg/L, *in vitro* exposure to GEN at a concentration of 4 mg/L had little effect on bacterial killing (Ruppen et al., 2016a). Similarly, and together with the rapid drop in drug levels observed in our TCF, it is possible that the GEN MIC, minimal bactericidal concentration (MBC), or MBEC increases with exposure to insufficient GEN concentration (i.e., development of tolerance). This theory is, however, challenging to prove since methods of susceptibility testing and MIC cut-off values for GEN susceptibility have not been established. In addition, testing must be performed with GBS dislodged from coverslips after sonication (i.e., returning the biofilm to planktonic bacteria), making the interpretation of results difficult. Notably, our hypothesis was tested *in vivo* with only one GBS isolate. Another testing limitation involves the interpretation of PK studies in a condition without infection and a PEN-dosing-free interval of 12 h due to ethical restrictions. Despite this, PK results indicated that PEN levels were above the MIC during the entire experimental period. GEN dosing was extrapolated from serum studies in humans despite a faster plasma elimination half-time in rats, although the GEN peak serum level (C_max_) is comparable between rats and humans (Tran Ba Huy et al., 1986). The criteria for the definition of synergism are arbitrary. Nonetheless, no impressive difference was seen when we compared the bacterial loads after PEN treatment with those after PEN–GEN treatment, irrespective of this definition.

## Conclusion

Our investigations in an experimental model of GBS foreign-body infection did not show considerable added value of systemically administered GEN as an adjunct to PEN for killing two different growth forms of sessile GBS. A possible explanation for this observation may be – in comparison to serum – the low GEN concentration in the extravascular compartment. The translation of these findings into clinical practice requires further studies with a higher number of strains. If GEN is used, the results of this study point toward the combination of systemic PEN plus local high-dose GEN in a slow-release drug formulation. This combination has a potential application in orthopedic device-associated infections caused by GBS.

## Author Contributions

CR performed the study, conducted the vast majority of experiments, and co-wrote the manuscript. TM and LD measured antibiotic concentration levels and approved the final version of the manuscript. DG and SL contributed considerably to the animal experiments, co-wrote the manuscript, and approved the final version of the manuscript. CEH and OM contributed to the study design of the animal experiments, performed a pilot study, and approved the final version of the manuscript. PS developed the study design, was responsible for the project, co-wrote the manuscript, and approved the final version.

## Conflict of Interest Statement

The authors declare that the research was conducted in the absence of any commercial or financial relationships that could be construed as a potential conflict of interest.
