# Supplementary material for: Is Penicillin Plus Gentamicin Synergistic Against Sessile Group B Streptococcal Isolates? An in Vivo Study With an Experimental Model of Foreign-Body Infection
Source: Front Microbiol. 2018 May 15;9:919. doi: 10.3389/fmicb.2018.00919 (PMC5962661; doi:10.3389/fmicb.2018.00919)
Supplement: Supplementary file 1 [file Data_Sheet_1.DOC]

**Legend to the Supplementary files 2.** Procedures including animal research. The number in brackets reflects the numbers of animals used for the experiment.

**Figure S2 A.** Graphical time schedule illustrating arrival of animals in the laboratory, surgical procedure (i.e., implantation of tissue cages) and sterility test of the tissue cage fluid.

**Figure S2 B.** Graphical time schedule illustrating pharmacokinetic studies after administration of systemic penicillin, systemic gentamicin and local gentamicin.

**Figure S2 C.** Graphical time schedule illustrating treatment protocols in the 4-arm study. The flash sign indicates the infection day (i.e., after sterility test, see also Figure S2 A). After day 3 and 6 of treatment, tissue cage fluid and coverslip was analysed for bacterial load in CFU/mL (indicated with a yellow star on day 25 and 28 of the upper time line). Each arrow indicates administration of a corresponding dose of antibiotics. Penicillin (PEN) is illustrated in blue, Gentamicin (GEN) in red, and sodium chloride (NaCl; control) in green. CV; coverslip.

The number in brackets in left column reflects the numbers of animals at the infection time point.

* Time point of euthanization and coverslip recovery from animals (number of animals euthanized, *a=4, *b=3 *c=3, *d=2). Experiments were continued with the corresponding number of animals.
