# Supplementary figures and images for: Is Penicillin Plus Gentamicin Synergistic Against Sessile Group B Streptococcal Isolates? An in Vivo Study With an Experimental Model of Foreign-Body Infection
Source: Front Microbiol. 2018 May 15;9:919. doi: 10.3389/fmicb.2018.00919 (PMC5962661; doi:10.3389/fmicb.2018.00919)

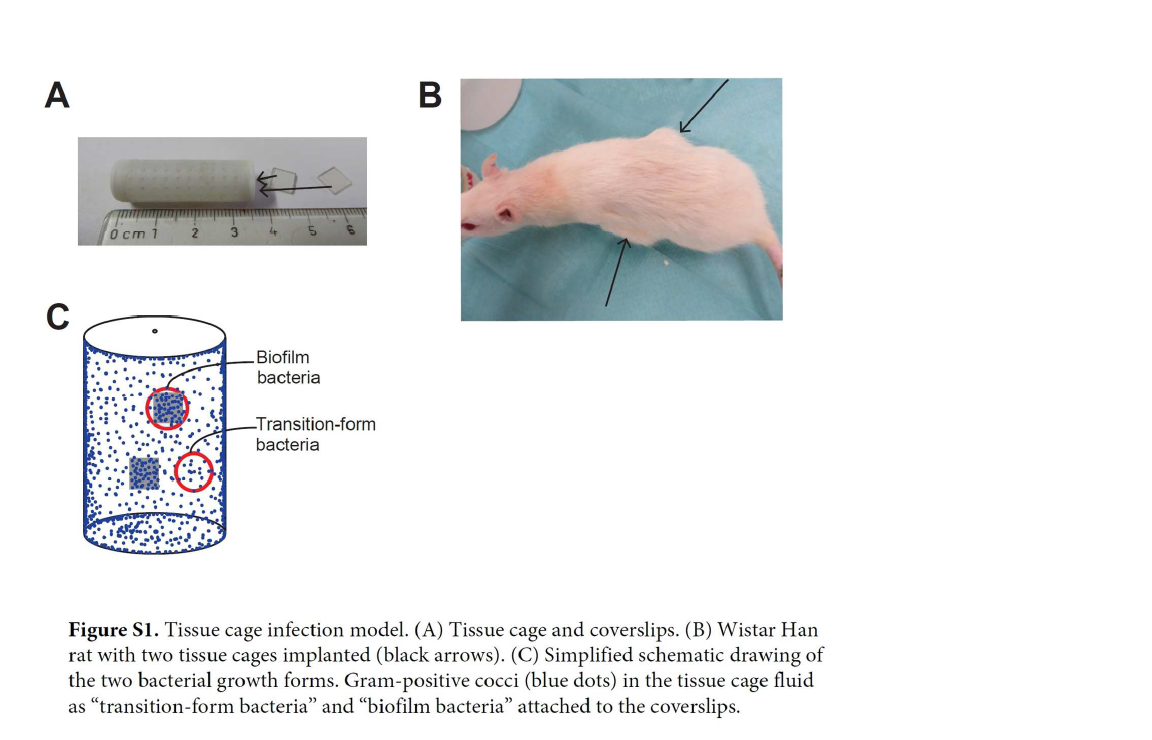

Supplement: Supplementary file 2 [file Image_1.TIF]

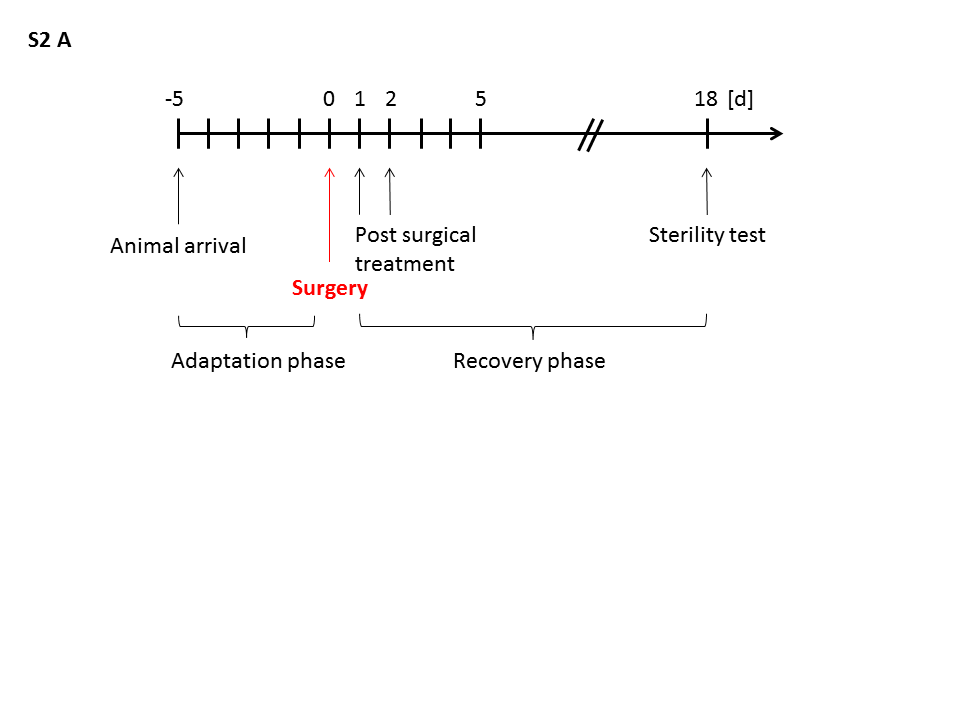

Supplement: Supplementary file 3 [file Image_2.TIF]

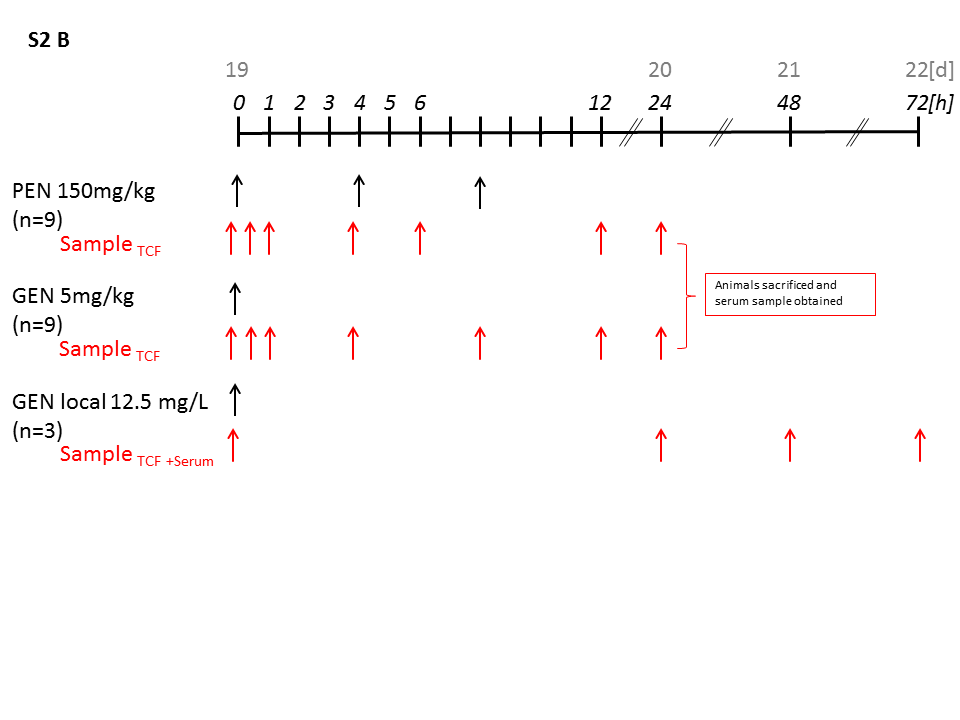

Supplement: Supplementary file 4 [file Image_3.TIF]

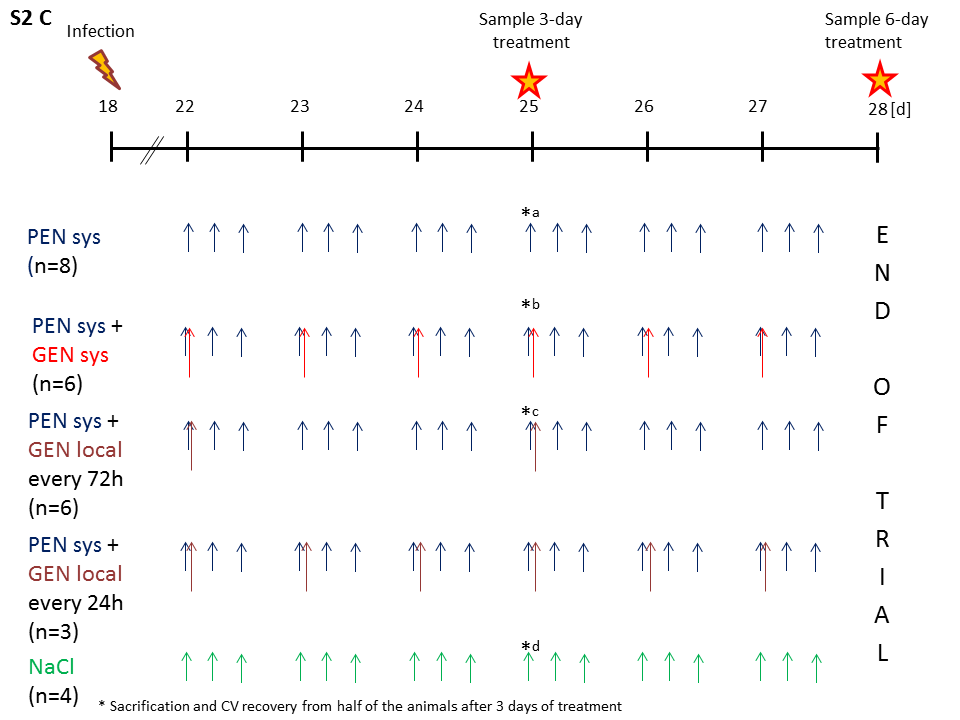

Supplement: Supplementary file 5 [file Image_4.TIF]
